# Reduced Bone Regeneration in Rats With Type 2 Diabetes Mellitus as a Result of Impaired Stromal Cell and Osteoblast Function—A Computer Modeling Study

**DOI:** 10.1002/jbm4.10809

**Published:** 2023-10-02

**Authors:** Mahdi Jaber, Lorenz C Hofbauer, Christine Hofbauer, Georg N Duda, Sara Checa

**Affiliations:** ^1^ Julius Wolff Institute at Berlin Institute of Health, Charité—Universitätsmedizin Berlin Berlin Germany; ^2^ Department of Medicine III and Center for Healthy Aging Technische Universität Dresden Dresden Germany; ^3^ BIH Center for Regenerative Therapies BIH at Charité ‐ Universitätsmedizin Berlin Berlin Germany

**Keywords:** BIOENGINEERING, BIOMECHANICS, DISEASES AND DISORDERS OF/RELATED TO BONE, OSTEOBLAST, STROMAL/STEM CELLS

## Abstract

Bone has the fascinating ability to self‐regenerate. However, under certain conditions, such as type 2 diabetes mellitus (T2DM), this ability is impaired. T2DM is a chronic metabolic disease known by the presence of elevated blood glucose levels that is associated with reduced bone regeneration capability, high fracture risk, and eventual non‐union risk after a fracture. Several mechanical and biological factors relevant to bone regeneration have been shown to be affected in a diabetic environment. However, whether impaired bone regeneration in T2DM can be explained due to mechanical or biological alterations remains unknown. To elucidate the relevance of either one, the aim of this study was to investigate the relative contribution of T2DM‐related alterations on either cellular activity or mechanical stimuli driving bone regeneration. A previously validated in silico computer modeling approach that was capable of explaining bone regeneration in uneventful conditions of healing was further developed to investigate bone regeneration in T2DM. Aspects analyzed included the presence of mesenchymal stromal cells (MSCs), cellular migration, proliferation, differentiation, apoptosis, and cellular mechanosensitivity. To further verify the computer model findings against in vivo data, an experimental setup was replicated, in which regeneration was compared in healthy and diabetic after a rat femur bone osteotomy stabilized with plate fixation. We found that mechanical alterations had little effect on the reduced bone regeneration in T2DM and that alterations in MSC proliferation, MSC migration, and osteoblast differentiation had the highest effect. In silico predictions of regenerated bone in T2DM matched qualitatively and quantitatively those from ex vivo μCT at 12 weeks post‐surgery when reduced cellular activities reported in previous in vitro and in vivo studies were included in the model. The presented findings here could have clinical implications in the treatment of bone fractures in patients with T2DM. © 2023 The Authors. *JBMR Plus* published by Wiley Periodicals LLC on behalf of American Society for Bone and Mineral Research.

## Graphical abstract

Bone has the fascinating ability to self‐regenerate; however, with type 2 diabetes mellitus (T2DM), this ability is impaired. Whether impaired bone regeneration in T2DM can be explained due to mechanical or biological alterations remains unknown. We found that mechanical alterations had little effect on the reduced bone regeneration in T2DM and that alterations in MSC proliferation, MSC migration, and osteoblast differentiation had the highest effect.

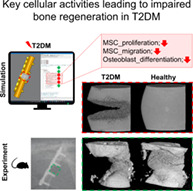

## Introduction

Bone has the fascinating ability to self‐regenerate. However, under certain conditions, such as in type 2 diabetes mellitus (T2DM), this ability is impaired.^(^ ^1^ ^)^ T2DM is a chronic metabolic disease known by the presence of elevated blood glucose levels that in addition to its well‐known association with cardiovascular disease, retinal disease, kidney disease, and polyneuropathy^(^ ^2^ ^)^ is associated with 40% to 70% increased risk for fractures,^(^ ^3^ ^)^ delayed healing by nearly 90%,^(^ ^4^ ^)^ and risk of fracture healing complications such as non‐union or delayed healing.^(^ ^5^ ^)^

Alterations in several cellular processes have been associated with impaired bone regeneration in T2DM. For instance, in T2DM, decreased bone regeneration has been linked to suppressed angiogenesis and osteogenesis.^(^ ^6^ ^)^ More specifically, osteoblast differentiation and proliferation were shown to be highly inhibited in the presence of diabetic environment.^(^ ^7^, ^8^, ^9^ ^)^ In an in vivo study, osteoblast differentiation markers such as ALP activity, RUNX2, OCN, and OPN showed highly reduced expression in T2DM rats during bone regeneration.^(^ ^1^ ^)^ Moreover, several cellular activities related to mesenchymal stromal cells (MSCs) have been shown to be inhibited in a diabetic environment. For example, Wang and colleagues^(^ ^10^ ^)^ reported that diabetes significantly hindered the migration of MSCs.^(^ ^10^ ^)^ Also, reduced MSC proliferation was observed under hyperglycemic conditions.^(^ ^11^, ^12^, ^13^ ^)^ Furthermore, the diabetic environment significantly decreases the MSC population and viability.^(^ ^11^ ^)^ In addition, mechanosensitivity of osteocytes has also been found to be altered in the presence of hyperglycemic environments, having a negative impact on bone formation.^(^ ^14^ ^)^ So far, it is known that many cellular activities involved in bone regeneration are altered in a T2DM environment; however, the relative contribution of these alterations at the cellular level on the cascades of bone regeneration are not well understood.

In addition to biological alterations, T2DM is known to affect the geometrical and mechanical properties of bone.^(^ ^15^ ^)^ Thinner cortical thickness,^(^ ^16^ ^)^ increased porosity, and lower bone volume to total volume^(^ ^17^ ^)^ have been reported in T2DM bones in rat models. Another study showed that T2DM in rats detrimentally affected cancellous and cortical geometry, which they attributed to explain low bone formation and high bone resorption.^(^ ^18^ ^)^ In addition, reduced bone stiffness, yield load, post‐yield energy, maximum load, and apparent modulus have been reported for T2DM bone tissue samples.^(^ ^19^, ^20^, ^21^ ^)^ Lastly, T2DM leads to an increased body weight in rats,^(^ ^16^ ^)^ which will translate into higher musculoskeletal loads at the fracture site. Up to now, whether impaired bone regeneration in T2DM is mainly driven by its cell‐biological alterations across various cell types or if the structural and mechanosensitive alterations dominate the pathophysiology of impaired bone regeneration in T2DM remains largely unknown.

Although it remains challenging in experimental approaches to determine the impact of a given mechanical or biological factor on the overall bone regeneration process, in silico approaches may help here. Understanding how T2DM‐related alterations contribute to impaired bone regeneration and the underlying mechanisms is vital to the development of effective approaches for accelerating bone healing in T2DM conditions. Computer modeling has the capacity to carve out underlying mechanisms of T2DM bone regeneration and formulate new hypotheses on the role of various factors affecting healing as well as their interdependencies, which are way too complex, ethically questionable, and time consuming to be done by other approaches. Specific computer models have been previously developed, validated, and applied to predict bone healing in various in vivo preclinical experiments.^(^ ^22^, ^23^, ^24^, ^25^, ^26^, ^27^, ^28^ ^)^ These models simulate the dynamics of the tissue formation process and allow to predict, even across a time course, the distribution patterns characteristic for tissues within a healing volume. These approaches allow the identification of how both mechanical environments and cellular activity determine the healing outcome. So far, such validated computer modeling approaches have not included systemic impairment of healing that are both affecting the cellular as well as the structural or mechanical constrains of healing. In this regard, T2DM may serve as a good model to investigate whether mechanostructural or cell‐biological alterations associated with T2DM dominate the pathological alterations found in bone regeneration.

The aim of this study was to investigate the relative contribution of T2DM‐related alterations on cell‐biological activity or mechanostructural alterations to impaired bone regeneration. To achieve this aim, a previously validated computer model of bone regeneration was used where bone healing predictions were compared with histological sections from in vivo experiments at several time points post‐surgery investigating the effect of fixation stiffness, species, and aging.^(^ ^22^, ^25^ ^)^ In this study, this computer model was further developed to include T2DM‐related alterations. Computer model predictions were compared with previously published ex vivo data of bone regeneration within a mechanically stabilized rat femoral osteotomy. Parametric analyses were carried out to identify the most important factors contributing to the impairment in the bone healing outcome in T2DM.

## Materials and Methods

### Simulated in vivo experiment

**Fig. 1:**
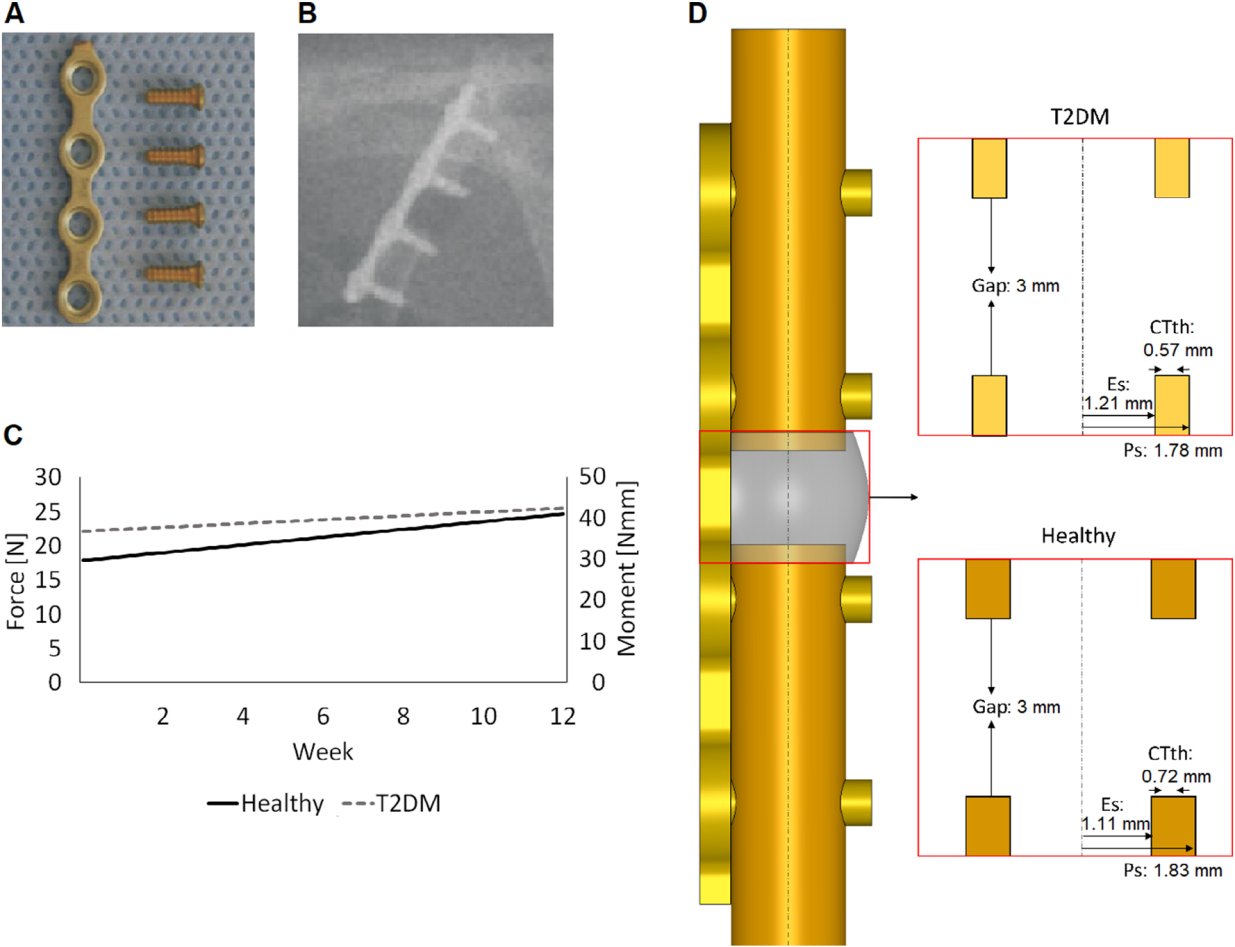
(*A*) Four‐hole plate with screws. (*B*) Postoperative X‐ray with plate and defect. (*C*) Applied compression force and bending moment during bone regeneration. (*D*) CAD model of the rat femoral osteotomy (height: 30 mm) stabilized with a plate. Cross sections of the callus for both healthy and type 2 diabetes mellitus (T2DM) models are shown with the red borderlines. Ps = periosteal radius; Es = endosteal radius; CTth = cortical thickness.^(^ ^1^, ^16^ ^)^

A previously described in vivo study^(^ ^1^ ^)^ was used to compare in silico predictions to in vivo observations of bone healing in healthy and T2DM rats. Details of the experimental study design are only briefly described here. Two groups of rats, diabetic (*n* = 6) and non‐diabetic (*n* = 6), underwent a 3‐mm cross‐sectional subcritical defect at the midshaft in the left femur at the age of 9 weeks (Fig. 1*A*). The osteotomy was held in place using a four‐hole plate (Stryker, Hamburg, Germany) and fixed with four screws (Fig. 1*B*). The initial and final body weight of the non‐diabetic and diabetic rats was 367 g and 296 g and 423 g and 409 g, respectively.^(^ ^1^ ^)^ Microcomputed tomography (μCT) analysis of the left femur was performed 12 weeks post‐mortem. Imaging was performed using Synchrotron micro‐computed tomography (SRmicroCT) device. Voxel resolution of 20 microm (SRmicroCT: 9 microm) and X‐ray energy of 70 keV (SRmicroCT: 55 keV) were used. Bone healing outcome after 12 weeks was quantified as the relative amount of newly formed bone relative to the area of the cortices.

### In silico bone regeneration model

A previously described and experimentally validated bone regeneration computer model was adapted^(^ ^25^ ^)^ to investigate bone regeneration in T2DM. The computer model combined finite element (FE) analysis, to determine the mechanical environment within the healing region, and an agent‐based model (ABM) describing the biological processes taking place during bone regeneration at the cellular level.^(^ ^25^ ^)^

#### Finite element model

Finite element (FE) computer models of the experimental rat osteotomies were created to determine the mechanical strains induced within the healing region during the healing process. The models included the femur, the medullary cavity, the plate, and the callus region (Fig. 1*D*). Because body weight and bone dimensions were different in diabetic and healthy rats (Fig. 1 *C*, *D*), two representative finite element models, one of each group, were built based on the experimental data.^(^ ^16^ ^)^ The femur was idealized as a hollow cylinder of cortical bone filled with marrow tissue. A 3‐mm opening was transversely created in the middle of the bone to simulate the fracture gap. The fracture opening and its surrounding area were included in a callus domain. After the experimental setup, the osteotomized bone model was then stabilized with a plate and four screws (Fig. 1).

FE models were built using commercial software ABAQUS/Standard 2019 (Simulia, Dassault Systemes, Vélizy‐Villacoublay, France). The model was meshed using three‐dimensional quadratic tetrahedral elements (C3D10MP) with an average mesh size of 0.50 mm for the whole model except for the callus region, where it had an average mesh size of 0.2 mm.

All biological tissues were modeled as poroelastic materials with properties given in Table 1. The plate and the nails were considered linear elastic with material properties of titanium (E = 110 GPa, v = 0.3).

**Table 1:** Tissue Material Properties were adapted from Checa et al.,^(^ ^25^ ^)^ except for the cortical bone Young's modulus, which was adapted from Hamann et al.^(^ ^16^ ^)^

	Granulation tissue	Fibrous tissue	Cartilage	Immature bone	Mature bone	Cortical bone	Bone marrow
Young's modulus (MPa)	0.2	2	10	1000	5000	8660 (healthy)	2
						6610 (T2DM)	
Permeability (m^4^/Ns.10^−14^)	1	1	0.5	10	37	0.001	1
Poisson's ratio	0.167	0.167	0.3	0.3	0.3	0.3	0.167
Bulk modulus grain (MPa)	2300	2300	3700	13,940	13,940	13,920	2300
Bulk modulus fluid (MPa)	2300	2300	2300	2300	2300	3200	2300

Compression and bending loads were applied as previously reported.^(^ ^29^ ^)^ Because diabetic and non‐diabetic rats had different body weights, different loads were applied to each FE model. In addition, because the body weights of the rats increase with time,^(^ ^1^ ^)^ this increase is adapted to the loads increase accordingly. Briefly, in the proximal bone end of the non‐diabetic model, a compression load of 17.42 N was initially applied in combination with a bending moment of 31.07 Nmm reaching 24.07 N and 42.93 Nmm at the end of the regeneration process, respectively, whereas in the diabetic model, a compression load of 21.60 N was initially applied in combination with a bending moment of 38.52 Nmm, reaching 24.89 N and 44.40 Nmm at the end of the regeneration, respectively (Fig. 1*C*). Boundary conditions were applied on the other bone end to restrain the movement in all directions.

#### Agent‐based modeling approach

An agent‐based computer modeling approach was selected and implemented using C++, where the space occupied by the regenerating tissue region was discretized into a 3D grid (spacing 10 μm). Each of the grid positions within the healing region represented a potential space a cell could occupy. The following cell phenotypes were included: MSCs, fibroblasts, chondrocytes, immature osteoblasts, and mature osteoblasts. The model simulates cellular processes including migration, proliferation, differentiation, and apoptosis. Informed by previous work, cell differentiation of MSCs into osteoblasts, chondrocytes, or fibroblasts was simulated to be influenced by the local mechanical stimuli within the healing region.^(^ ^30^ ^)^

To simulate the invasion of MSCs from the marrow cavity and periosteum, 30% of the grid positions along the periosteum and marrow cavity were initially seeded with MSCs.^(^ ^25^ ^)^ Cells were simulated to produce the corresponding extracellular matrix (osteoblasts: bone, chondrocytes: cartilage, and fibroblasts: fibrous tissue), that was implemented as a change in the mechanical properties of the tissue in the FE model (Section 2.2.1). Each element property was determined using a rule of mixtures^(^ ^31^ ^)^ to account for different tissues being present in the same element. In addition, each element property was averaged over the last 10 iterations to account for the delay in actual extracellular matrix maturation.^(^ ^27^ ^)^

The cellular processes incorporated into the computer model exhibit partial randomness, contributing to the overall variability observed in the simulations. Stochastic behaviors, including migration, proliferation, differentiation, and apoptosis, are incorporated in the model. For example, during MSC migration, the direction of cell movement is randomly determined among available positions, considering neighboring free agents. Similarly, random searches for neighboring free agents occur during cell proliferation, differentiation, and death processes. These stochastic components enable the exploration of diverse outcomes and account for inherent variability within the model.

### Modeling T2DM‐related alterations in cellular activity

To develop the model of bone regeneration in T2DM, a literature review was performed to collect reported alterations in cellular function and their levels due to T2DM. Reported alterations on cellular activities involved in bone regeneration caused by T2DM are summarized in Table 2.

**Table 2:** Summary of reported alterations of cellular activities involved in bone regeneration caused by Type 2 Diabetes Mellitus

Cellular activity	Level of alteration (% to healthy)	Reference
MSC migration	60% decrease	Wang et al.^(^ ^10^ ^)^
Fibroblast migration	75% decrease	Lerman et al.^(^ ^32^ ^)^
No. of MSCs in the bone marrow	33% decrease	Kim et al.^(^ ^11^ ^)^
Chondrocyte differentiation	50% decrease	Roldan et al.^(^ ^33^ ^)^
Osteoblast differentiation	50% decrease	Kočí et al.,^(^ ^7^ ^)^ Li et al.,^(^ ^8^ ^)^ Turner et al.^(^ ^9^ ^)^
MSC proliferation	65%–70% decrease	Kim et al.,^(^ ^11^ ^)^ Marycz et al.,^(^ ^12^ ^)^ Stolzing et al.^(^ ^13^ ^)^
Fibroblast proliferation	50% decrease	Desta et al.^(^ ^34^ ^)^
Chondrocyte proliferation	40% decrease	Kayal et al.^(^ ^35^ ^)^
Osteoblast proliferation	50% decrease	Li et al.^(^ ^8^ ^)^
MSC apoptosis	67% increase	Liu et al.^(^ ^36^ ^)^
Fibroblast apoptosis	600% increase	Desta et al.^(^ ^34^ ^)^
Chondrocyte apoptosis	100% increase	Aeimlapa et al.^(^ ^37^ ^)^
Osteoblast apoptosis	50% increase	Sun et al.^(^ ^38^ ^)^
Cellular mechanosensitivity	‐	Parajuli et al.^(^ ^14^ ^)^

Abbreviation: MSC = mesenchymal stromal cell.

### Modeling healthy and diabetic bone regeneration processes

Based on the literature summarized in Table 2, two different bone regeneration models were created: one representative of the healthy group (healthy) and one representative of the group with T2DM (T2DM). The healthy model adopted model parameters of previous studies that showed the prediction of bone healing in uneventful situations.^(^ ^25^ ^)^ The T2DM model adopted model parameters that were scaled relative to the healthy ones, according to the values reported in Table 2. Table 3 summarizes the parameter values for both the healthy and the T2DM bone regeneration models. Cellular mechanosensitivity in the healthy model followed the mechanical stimuli ranges according to Checa and colleagues.^(^ ^25^ ^)^ The cellular mechanosensitivity was reduced in the diabetic model by shifting the ranges of mechanical stimuli driving MSC differentiation into fibroblasts, chondrocytes, and osteoblasts to higher levels and including a lazy zone (Supplemental Materials and Methods) after experimental observations of reduced cellular mechanosensitivity (Table 2).

**Table 3:** Model parameters for the healthy and T2DM bone regeneration models

Cellular activity	Levels	
	Healthy	T2DM
Initial MSC density: periosteum	30%	19.5%
Initial MSC density: bone marrow	30%	19.5%
MSC migration rate	30 μ/h	12 μ/h
Fibroblast migration rate	30 μ/h	7.5 μ/h
Fibroblast differentiation rate	0.3/d	0.15/d
Chondrocyte differentiation rate	0.3/d	0.15/d
Osteoblast differentiation rate	0.3/d	0.15/d
MSC proliferation rate	0.6/d	0.21/d
Fibroblast proliferation rate	0.55/d	0.275/d
Chondrocyte proliferation rate	0.2/d	0.12/d
Osteoblast proliferation rate	0.3/d	0.15/d
MSC apoptosis rate	0.05/d	0.0835/d
Fibroblast apoptosis rate	0.05/d	0.3/d
Chondrocyte apoptosis rate	0.1/d	0.2/d
Osteoblast apoptosis rate	0.16/d	0.24/d
Cellular mechanosensitivity	Supplemental data 1	
Plate‐surface guidance enhancement	10%	Healthy rates

Abbreviations: MSC = mesenchymal stromal cell; T2DM = type 2 diabetes mellitus.

**Fig. 2:**
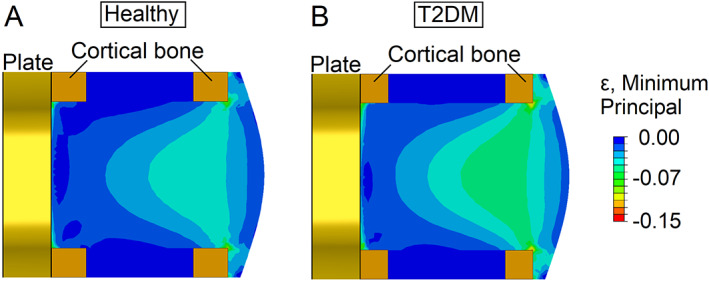
Minimum principal compressive strains in the mid cross section across the callus, immediately after surgery in (*A*) healthy and (*B*) type 2 diabetes mellitus (T2DM). The T2DM callus exhibited higher strains when compared with the healthy one.

In addition, experimentally, a considerably higher amount of bone formed closer to the fixation plate compared with the opposite site due to increased osteogenic capacity of the plate‐treated surface^(^ ^39^, ^40^, ^41^, ^42^ ^)^ (Fig. 2). Therefore, in this study, the role of plate‐surface guidance on bone formation was investigated. In the healthy and T2DM models, all the cellular activities happening close to the plate (30 micrometers far from the plate) were increased (Table 3).

### Analyzing the influence of mechanostructural parameters on bone regeneration

To investigate the effect of T2DM‐related alterations in mechanostructural parameters (thinner cortical thickness, reduced cortical bone stiffness, and higher body weight) found in diabetic rats on the bone healing outcome,^(^ ^16^ ^)^ first the mechanical environment within the callus region of both the healthy and the diabetic models were compared. Thereafter, the model with T2DM mechanostructural parameters was run using cell‐biological activity rates from the healthy group.

### Parametric analysis of the effect of T2DM‐related cell‐biological alterations impacting bone healing

To determine the relative contribution of T2DM‐related alterations in cellular activity to bone healing, a parametric study based on design of experiments was performed. The influence of 16 parameters related to cellular behavior on bone healing predictions was investigated. Two levels were assigned for each parameter: healthy (+1) and T2DM (−1).

Orthogonal arrays were used in a Plackett–Burman design^(^ ^43^ ^)^ to reduce the number of experiments necessary to investigate interactions between the parameters. Twenty‐run Plackett–Burman design was chosen where 20 different experiments were designed to investigate the contribution of each of the 16 parameters to T2DM‐related bone‐healing alterations. The design of experiments requires 19 parameters, whereas the investigated cellular parameters were 16. To tackle this, three additional “dummy” parameters were randomly used to complete the design of experiments. These dummy parameters had no physical meaning.^(^ ^44^ ^)^ Each one of the experiments was characterized by a defined combination of parameters associated with their healthy (+1) or T2DM level (−1) (Table 4).

**Table 4:** Orthogonal matrix with the 20 experimental conditions as combination of the parameters on two levels

Experiments/ Parameters	1	2	3	4	5	6	7	8	9	10	11	12	13	14	15	16	17	18	19
**1**	−1	−1	−1	−1	−1	−1	−1	−1	−1	−1	−1	−1	−1	−1	−1	−1	−1	−1	−1
**2**	1	−1	1	1	−1	−1	−1	−1	1	−1	1	−1	1	1	1	1	−1	−1	1
**3**	1	1	−1	1	1	−1	−1	−1	−1	1	−1	1	−1	1	1	1	1	−1	−1
**4**	−1	1	1	−1	1	1	−1	−1	−1	−1	1	−1	1	−1	1	1	1	1	−1
**5**	−1	−1	1	1	−1	1	1	−1	−1	−1	−1	1	−1	1	−1	1	1	1	1
**6**	1	−1	−1	1	1	−1	1	1	−1	−1	−1	−1	1	−1	1	−1	1	1	1
**7**	1	1	−1	−1	1	1	−1	1	1	−1	−1	−1	−1	1	−1	1	−1	1	1
**8**	1	1	1	−1	−1	1	1	−1	1	1	−1	−1	−1	−1	1	−1	1	−1	1
**9**	1	1	1	1	−1	−1	1	1	−1	1	1	−1	−1	−1	−1	1	−1	1	−1
**10**	−1	1	1	1	1	−1	−1	1	1	−1	1	1	−1	−1	−1	−1	1	−1	1
**11**	1	−1	1	1	1	1	−1	−1	1	1	−1	1	1	−1	−1	−1	−1	1	−1
**12**	−1	1	−1	1	1	1	1	−1	−1	1	1	−1	1	1	−1	−1	−1	−1	1
**13**	1	−1	1	−1	1	1	1	1	−1	−1	1	1	−1	1	1	−1	−1	−1	−1
**14**	−1	1	−1	1	−1	1	1	1	1	−1	−1	1	1	−1	1	1	−1	−1	−1
**15**	−1	−1	1	−1	1	−1	1	1	1	1	−1	−1	1	1	−1	1	1	−1	−1
**16**	−1	−1	−1	1	−1	1	−1	1	1	1	1	−1	−1	1	1	−1	1	1	−1
**17**	−1	−1	−1	−1	1	−1	1	−1	1	1	1	1	−1	−1	1	1	−1	1	1
**18**	1	−1	−1	−1	−1	1	−1	1	−1	1	1	1	1	−1	−1	1	1	−1	1
**19**	1	1	−1	−1	−1	−1	1	−1	1	−1	1	1	1	1	−1	−1	1	1	−1
**20**	−1	1	1	−1	−1	−1	−1	1	−1	1	−1	1	1	1	1	−1	−1	1	1

*Note*: Healthy (+1) and type 2 diabetes mellitus (−1).

The bone volume predicted at 12 weeks by each of the 20 experiments was used to perform analysis of variance and evaluate the contribution of each parameter to the T2DM‐related alteration of bone healing. The sum of the squares (SS) was calculated for each parameter. The SS represented the contribution of each single parameter and was considered a measure of the “importance” of each parameter.

(1) $$\mathrm{SS}=\sum_{i=0}^{n}{\left(X_{i}-\bar{X}\right)}^{2}$$

where n is the total number of experiments, i is the given experiment, $X_{i}$ is the BV/TV% at the 12th week of a parameter for a given experiment i and $\bar{X}$ is the mean BV/TV% at the 12th week in all experiments.

The most influential parameters were then tested for their contribution to T2DM‐related alterations in bone regeneration. To achieve this, additional bone healing simulations were performed where all parameters were set to healthy levels except for the ones showing a strong contribution in the design of experiments.

### In silico experiments

A summary of the different in silico experiments conducted and the corresponding number of simulation runs for each experiment are provided in Table 5.

**Table 5:** Summary of the total number of in silico experiments performed in this study

Experiment description	No. of runs
Healthy with healthy rates without surface guidance	1
T2DM with healthy rates without surface guidance	1
T2DM with T2DM rates without surface guidance	1
Healthy with healthy rates with surface guidance	1
T2DM with T2DM rates with surface guidance	1
Parametric analysis (varying parameters)	20
Combination of top influential parameters	15
Top influential parameters with surface guidance	1
Total	41

Abbreviation: T2DM = type 2 diabetes mellitus.

### Output analysis: BV/TV % and μCT images at 12 weeks

Predicted bone tissue distribution after 12 weeks of healing in healthy and T2DM models was compared qualitatively with μCT images of bone healing in the experimental study.^(^ ^1^ ^)^

In addition, BV/TV % in the gap region was quantified for all simulations after 12 weeks and compared with the experimental data.^(^ ^1^ ^)^

## Results

### T2DM leads to higher post‐surgery mechanical strains within the healing region

At time 0 of the bone regeneration process, ie, immediately after surgery, average compressive principal strains within the callus region were higher in the T2DM model compared with the healthy model (Fig. 2). Higher strains were predicted intercortically in the medial side compared with the lateral side (plate side), both for T2DM and healthy (Fig. 2). A decreasing gradient of strains was observed from the medial to the lateral side between the intercortical regions, in both T2DM and healthy. Relatively low strains were predicted at the lateral side near the plate. In the intercortical region, in the lateral side, average strains within the callus were 0.3% and 0.23% in T2DM and healthy models, respectively. In the medial side, average strains within callus were 0.47% and 0.37% in T2DM and healthy models, respectively. Strain differences between the healthy and T2DM models were 30% in the lateral side and 27% in the medial side.

### T2DM‐related alterations in mechanostructural effects cannot explain impaired bone regeneration

**Fig. 3:**
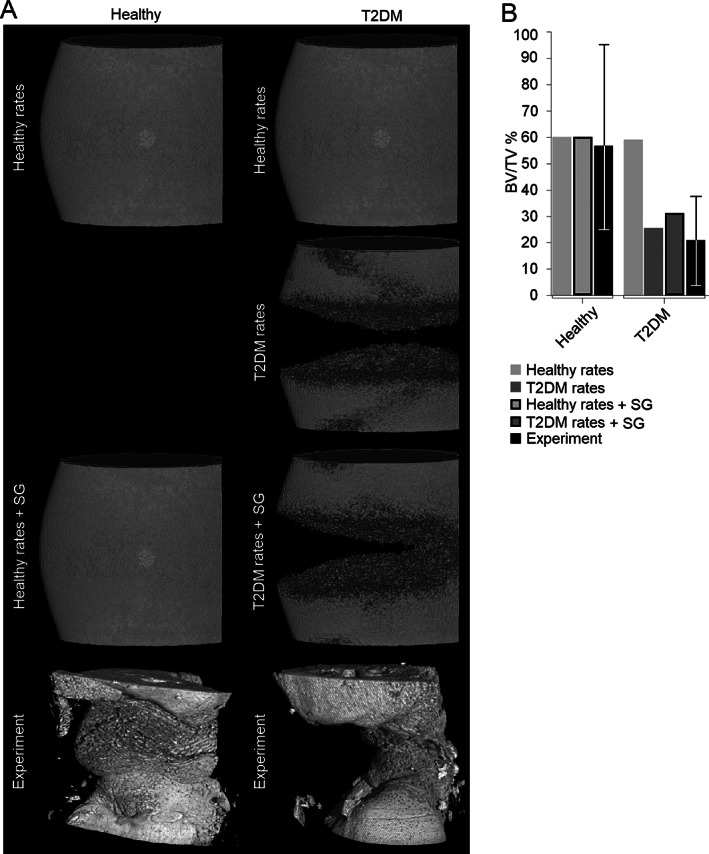
(*A*) Predicted bone formation with healthy and type 2 diabetes mellitus (T2DM) models assuming healthy cellular behavior, T2DM‐related altered cellular behavior, and T2DM‐related altered cellular behavior combined with plate‐surface guidance for bone formation. μCT images of regenerated bone in healthy and T2DM groups (experiment). (*B*) Experimental and predicted bone volume to total volume within the gap region (error bars represent the maximum and minimum amount of bone quantified in 10 samples for healthy and 7 samples for T2DM). The healthy bone model was not run using T2DM parameters because the objective was to investigate where mechanostructural alterations in T2DM could explain reduced bone formation. SG: Surface guidance.

When the previously validated bone healing algorithm for uneventful bone healing^(^ ^25^ ^)^ was used as a representative model for the healthy case, bone defect regeneration across the subcritical defect (3 mm) could be predicted (Fig. 3*A*). The computer model did not only match the experiment qualitatively but also quantitatively: Predicted bone volume to total volume was within the range of experimental data (healthy, experiment: 57%; simulation: 60%).

In the T2DM model, when the healthy rates (from Table 3) were used, the healing was similar to the healthy model after 12 weeks and was not able to explain experimentally observed alterations in bone healing outcome in T2DM animals (Fig. 3).

### Reduced MSC migration, MSC proliferation, and osteoblast differentiation can explain impaired bone regeneration in T2DM

Simulation of the bone healing process including experimentally reported T2DM‐related alterations in cellular activity resulted in impaired healing, ie, non‐union after 12 weeks (Fig. 3). The predicted bone volume to total volume was considerably reduced in the T2DM compared with the healthy model (simulation, healthy: 60%; T2DM: 27%). Predicted regenerated bone in the T2DM model compared well with experimental data in terms of bone volume to total volume (T2DM, experiment: 21%; simulation: 27%). Bone distribution within the callus was, however, different because experimentally more bone was formed close to the plate that was not predicted by the model.

Simulation of plate‐surface guidance resulted in predicted bone formation after 12 weeks comparable to experimental observations, both qualitatively and quantitatively (simulation, T2DM + plate‐surface guidance: 32%). The T2DM + plate‐surface guidance model was able to predict bone formation at the plate surface, which resulted in bone bridging at the lateral side (plate side) and non‐union in the medial callus side (Fig. 3). When the plate‐surface guidance effect was added to the healthy model, no effect could be observed (Fig. 3).

The parametric analysis based on DoEs showed that T2DM‐related alterations in MSC proliferation, MSC migration, and osteoblast differentiation played the main role in T2DM alterations in bone regeneration. T2DM‐related alterations in MSC proliferation and migration had the highest effect on predicted bone formation outcome, followed by osteoblast differentiation (Table 6).

**Table 6:** Sum of the squares for each cellular parameter investigated with the T2DM bone regeneration model

Cell activity	SS—Median (SS)
Initial MSC density: periosteum	0
Initial MSC density: bone marrow	0
MSC migration rate	**0.04**
Fibroblast migration rate	0
Dummy	0
Fibroblast differentiation rate	0
Chondrocyte differentiation rate	0
Osteoblast differentiation rate	**0.08**
Dummy	0
MSC proliferation rate	**0.48**
Fibroblast proliferation rate	0
Chondrocyte proliferation rate	0
Osteoblast proliferation rate	**0.01**
Dummy	0
MSC apoptosis rate	0
Fibroblast apoptosis rate	0
Chondrocyte apoptosis rate	0
Osteoblast apoptosis rate	0
Cellular mechanosensitivity	0

*Note*: Median (SS) = 3.55.

Abbreviations: MSC = mesenchymal stromal cell; T2DM = type 2 diabetes mellitus.

**Fig. 4:**
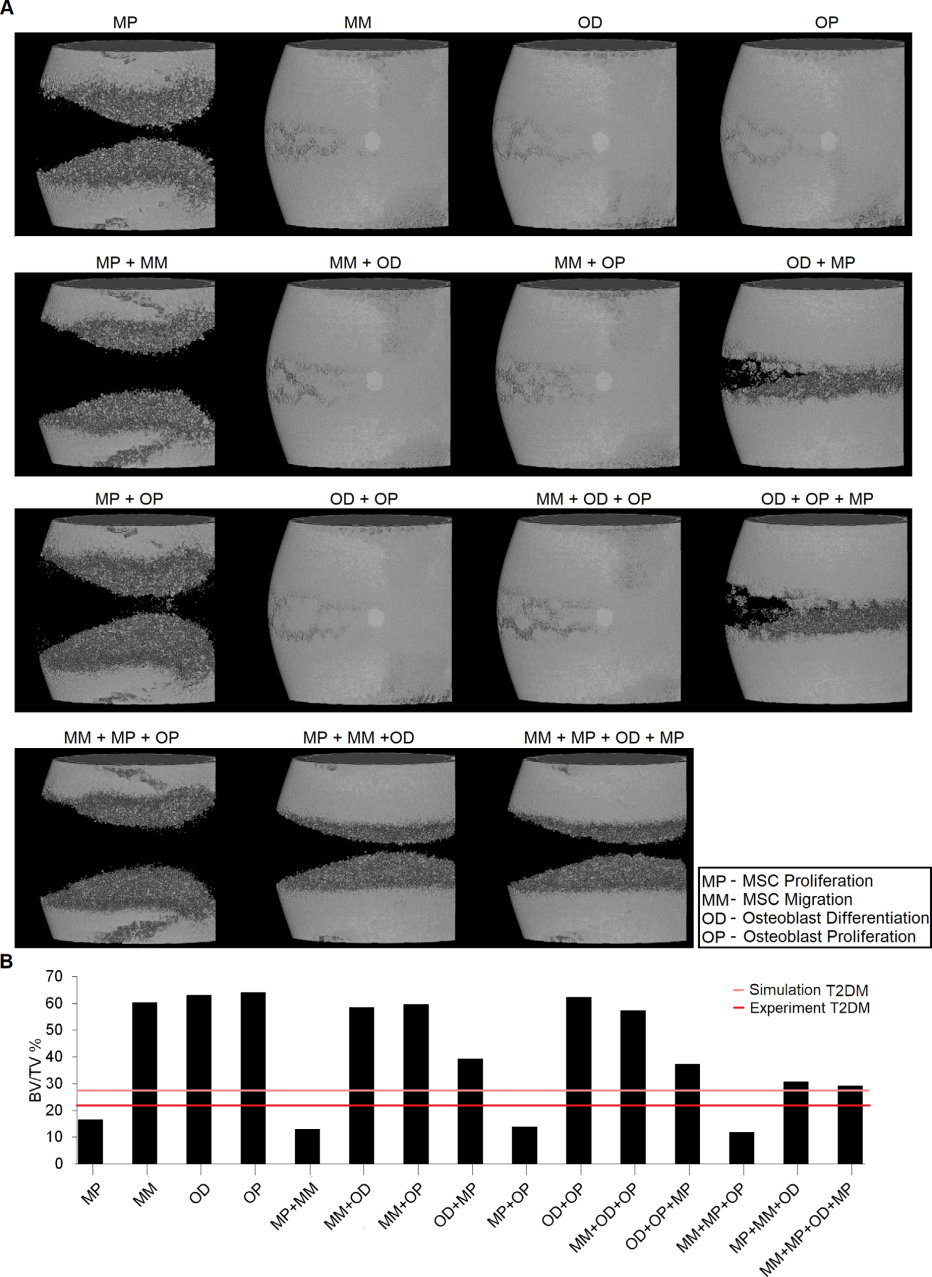
Bone healing predictions after 12 weeks for the type 2 diabetes mellitus (T2DM) model when only the most influential parameters were set to T2DM levels. The effect of the most influential parameters was investigated individually and in combination to investigate potential interactions. (*A*) μCT‐like image and (*B*) bone volume to total volume.

**Fig. 5:**
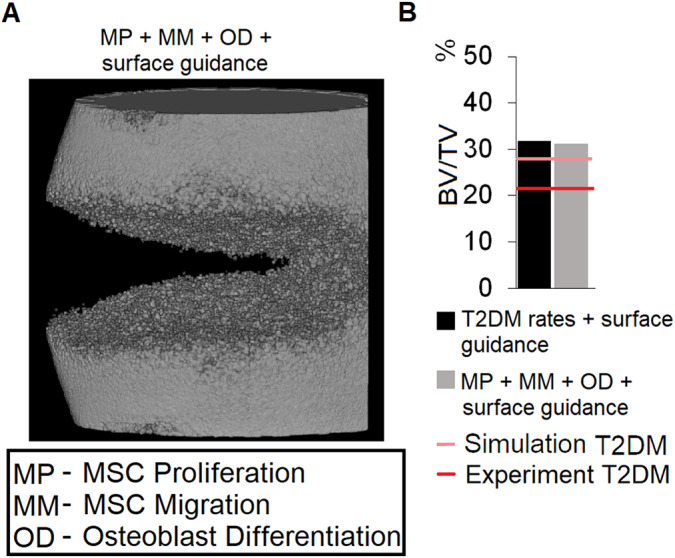
Bone healing prediction at 12 weeks when considering the type 2 diabetes mellitus (T2DM) model with altered mesenchymal stromal cell (MSC) migration, MSC proliferation, and osteoblast differentiation and plate‐surface guidance. (*A*) μCT‐like image and (*B*) bone volume to total volume.

Both qualitatively and quantitatively, T2DM‐related alterations in MSC proliferation, MSC migration, and osteoblast differentiation were able to explain experimentally observed alterations in bone healing outcome in T2DM animals (Fig. 4). Computer models of bone regeneration with T2DM‐related reduced levels in only these three cellular activities predicted non‐union after 12 weeks, as what was reported experimentally (Fig. 4). By adding plate‐surface guidance, predicted bone regeneration was similar to both the experiment and the predictions done with all altered parameters (Fig. 5).

## Discussion

From clinical and preclinical work, we are aware that T2DM impairs bone regeneration. Diverse cell‐biological and mechanostructural factors have been related to this impairment.^(^ ^7^, ^9^, ^10^, ^19^, ^20^, ^21^, ^32^, ^33^, ^45^ ^)^ However, the relative role of either the cell‐biological or the mechanostructural alterations associated with T2DM on bone regeneration remained so far unknown. In this study, using a combine, we developed a computer model of T2DM bone regeneration and compared its predictions to in vivo experimental data. Using this model, we investigated the relative contribution of T2DM‐related alterations on cell‐biological activity and mechanostructural properties to bone regeneration in rat models. To our surprise, we found that mechanostructural alterations had little effect on the reduced bone regeneration in rats with T2DM and that alterations in MSC proliferation, MSC migration, and osteoblast differentiation have the highest effect on impaired bone regeneration in rats with T2DM.

In this study, we first tested the ability of an existing computer model of bone regeneration that had been validated for uneventful bone healing^(^ ^25^, ^27^ ^)^ to predict bone regeneration within a subcritical defect in healthy rat.^(^ ^1^ ^)^ Our results show that mechanobiological computer models of uneventful bone regeneration are able to explain bone regeneration in a subcritical femoral rat bone osteotomy. For the given experimental setup investigated in this study, our simulations showed bone formation like that of the experiment both qualitatively and quantitatively.^(^ ^1^ ^)^ The adopted mechanobiological computer model of bone regeneration has already been able to explain bone healing outcomes in other scenarios where the computer model predictions of bone healing were compared with histological sections from in vivo experiments at several time points post‐surgery investigating the effect of fixation stiffness, species, and aging.^(^ ^22^, ^25^ ^)^ In this study, the computer model predictions in healthy and T2DM were validated by comparing the predicted bone tissue formation patterns to experimentally observed patterns measured using uCT and by comparing the BV/TV %.^(^ ^1^ ^)^ Here, for the first time, it was applied to a subcritical defect.

Although mechanostructural alterations are the result of altered biological function, we focused on unraveling their impact on the mechanical environment. This interplay between mechanostructural alterations and mechanical conditions provides valuable insights into impaired bone regeneration. To test whether mechanostructural alterations in T2DM alone can explain impaired bone regeneration found in T2DM, we simulated the thinner cortical thickness, higher body weight, and lower bone stiffness found in vivo in T2DM animals.^(^ ^16^ ^)^ Using the bone healing algorithm used in the healthy case^(^ ^25^ ^)^ but with the T2DM mechanostructural alterations, our simulations showed an overestimation of the bone formation after 12 weeks of healing. Therefore, our results show that mechanostructural alterations alone are not able to explain impaired bone regeneration in rats with T2DM and that biological factors are required to explain impaired bone regeneration.

Based on experimental observations of alterations in cellular function and their levels due to T2DM,^(^ ^7^, ^9^, ^10^, ^32^, ^33^, ^45^ ^)^ the model parameters were scaled relative to the healthy ones to simulate T2DM bone regeneration. After 12 weeks of healing, the simulation results compared well with in vivo results, quantitatively. Both in silico and in vivo impaired healing was observed.

Although computer predictions of bone regeneration in T2DM compared well with experimental data, the distribution of the bone (assessed by μCT) was different. More bone was observed experimentally at the side where the plate was located. The higher amount of bone forming at the plate side could only be explained by an increased cellular activity for cells located in the plate vicinity, which can be attributed to an increased osteogenesis due to plate‐surface treatment.^(^ ^39^, ^40^, ^41^, ^42^ ^)^ When plate‐surface guidance was added to the T2DM bone regeneration computer model, simulation results compared well both qualitatively and quantitatively to observed bone formation experimentally.^(^ ^1^ ^)^ Plate‐surface guidance, however, had no effect when added to the healthy bone regeneration computer model. This was expected because bone bridging was already observed before including this feature in the healthy model. The exact level of enhancement due to plate‐surface guidance remains unknown and could not be evaluated. Nonetheless, in the healthy model, cellular rate enhancements did not influence bone formation patterns, whereas in the T2DM model, matching healthy cellular activity levels explained the observed bone formation patterns.^(^ ^1^ ^)^

The parametric analysis results revealed that T2DM‐related alterations in MSC proliferation, MSC migration, and osteoblast differentiation have the highest influence on the experimentally observed impaired bone regeneration. A bone regeneration computer model with only these three parameters set to T2DM levels was enough to reproduce results obtained from the T2DM model that had all 16 parameters set to T2DM levels, both qualitatively and quantitatively, suggesting that T2DM‐impaired bone regeneration could be explained due to impairment in only these cellular activities. These results are in line with experimental in vivo data that showed, in T2DM bone healing, a downregulation of expression levels of genes (osteopontin, bone morphogenetic protein‐2, RUNX2, and osteocalcin) known to be associated with MSC proliferation, MSC migration, and osteoblast differentiation.^(^ ^46^, ^47^, ^48^, ^49^, ^50^, ^51^ ^)^ In this study, a 20‐run Plackett–Burman design with 19 parameters was employed, where 16 parameters were real and 3 dummy. The presence of dummy parameters allowed us to assess potential interaction effects among the parameters.^(^ ^52^ ^)^ If the impact of the dummy parameters would have been substantial, it would indicate the presence of significant interaction effects. However, dummy parameters showed a small effect.

This study has several limitations. Although our study emphasizes the downregulation of the three identified factors, namely MSC proliferation, MSC migration, and osteoblast differentiation, as key players on impaired bone regeneration in T2DM, we acknowledge that our model's sensitivity to other contributing factors may be limited. Processes like angiogenesis, advanced glycation, adipogenesis, and inflammation were not explicitly modeled; however, their contribution to altered cellular behavior was taken into account as a reduction in cellular activity rates as observed experimentally. Future research should explore the contribution of additional factors to impaired regeneration in T2DM. To determine the mechanical conditions within the healing region, a cylindrical geometry was used to approximate the geometry of the femur. Although it may not fully capture the intricate three‐dimensional architecture of the bone, the mechanical conditions within the healing region mainly depend on the diameter of the bone and the cortical thickness.^(^ ^53^ ^)^ Our study shows that mechanostructural alterations associated with T2DM in rats cannot explain the altered bone regeneration response; however, effects of diabetes on the bone mechanostructural parameters seem to be bigger in humans.^(^ ^54^ ^)^ The translation of these findings to the human situation remains to be investigated. Also, although cellular mechanoresponse has been reported to be altered with diabetes,^(^ ^14^ ^)^ the level of this alteration remains unknown. The exact alteration of cellular mechanoresponse for T2DM bone healing could not be evaluated. Nevertheless, reported alterations in the other cellular activities were sufficient to explain the altered bone response. Finally, bone healing predictions were only compared with in vivo μCT images of the healing outcome at only one time point (end of the healing process). Future studies should investigate the dynamics of healing and how it is altered in T2DM.

In summary, we identified key factors behind impaired bone regeneration in T2DM in rats using a combined in vivo/in silico approach, and, they illustrate a dominating role of the biological and to a lesser degree the mechanical impairments associated with T2DM. Nonetheless, analyzing the mechanostructural alterations caused by T2DM in patients and understanding their impact on the mechanical conditions during the healing process can provide valuable insights for optimizing treatment decisions related to fixation stability. Moreover, the reduced healing capacity in T2DM could be explained due to impaired MSC proliferation, MSC migration, and osteoblast differentiation. Focusing on these biological aspects could help to focus clinical improvements to target enhanced regeneration in T2DM clinical cases.

## Author Contributions

**Mahdi Jaber:** Conceptualization; data curation; formal analysis; investigation; methodology; validation; visualization; writing – original draft; writing – review and editing. **Lorenz Hofbauer:** Formal analysis; investigation; resources; validation. **Christine Hofbauer:** Data curation; formal analysis; investigation; validation. **Georg Duda:** Conceptualization; investigation; project administration; resources; supervision. **Sara Checa:** Conceptualization; formal analysis; funding acquisition; investigation; methodology; project administration; resources; supervision; validation; writing – original draft; writing – review and editing.

## Disclosures

The authors have no conflicts of interest to declare. All co‐authors have seen and agree with the contents of the article, and there is no financial interest to report. We certify that the submission is original work and is not under review at any other publication.

### Peer Review

The peer review history for this article is available at https://www.webofscience.com/api/gateway/wos/peer-review/10.1002/jbm4.10809.

## Supporting information


**Table S1.** Mechano‐regulation algorithms for progenitor cell differentiation. Healthy: (adapted from^25^) and T2DM: Estimated from^14^.
**Table S2.** BV/TV % at the end of the regeneration process (12th week) was predicted for each one of the 20 designed experiments and the sum of the squares for each parameter.
