# Supplementary material for: Reduced Bone Regeneration in Rats With Type 2 Diabetes Mellitus as a Result of Impaired Stromal Cell and Osteoblast Function—A Computer Modeling Study
Source: JBMR Plus. 2023 Oct 2;7(11):e10809. doi: 10.1002/jbm4.10809 (PMC10652174; doi:10.1002/jbm4.10809)
Supplement: Supplementary file 1 — Table S1. Mechano‐regulation algorithms for progenitor cell differentiation. Healthy: (adapted from25) and T2DM: Estimated from14. Table S2. BV/TV % at the end of the regeneration process (12th week) was predicted for each one of the 20 designed experiments and the sum of the squares for each parameter. [file JBM4-7-e10809-s001.pdf]

Supplementary Data

Supplementary Table 1 Mechano-regulation algorithms for progenitor cell differentiation. Healthy: *(adapted from (Checa et al. 2011)) and T2DM: Estimated from (Parajuli et al., 2011)*

Stimulus: S g: shear strain, v: fluid velocity a = 0.0375a, b = 0.003 mm/s	Bone resorption	Mature osteoblast	Immature osteoblast	Chondrocyte	Fibroblast
Healthy	S<=0.01	0.01<S<=2.53	2.53<S<=3	3<S<=5	S>5
T2DM	S<=0.01	0.02<S<=2.54	2.54<S<=3.01	3<S<=5.01	S>5.01

Supplementary Table 2. BV/TV % *at the end of the regeneration process (12th week) was predicted for each one of the 20 designed experiments and the sum of the squares for each parameter*

Experiment	BV/TV %
E1	26
E2	36.2
E3	53
E4	38.5
E5	33
E6	10
E7	13.5
E8	60
E9	56.5
E10	13.7
E11	58.5
E12	56.5
E13	10.5
E14	13.5
E15	57
E16	38.7
E17	52.7
E18	41.2
E19	30.8
E20	61.7
